# Evaluation of lightweight fibreglass heel casts in the management of ulcers of the heel in diabetes: study protocol for a randomised controlled trial

**DOI:** 10.1186/1745-6215-15-462

**Published:** 2014-11-26

**Authors:** William Jeffcoate, Frances Game, Patricia Price, Ceri Phillips, Vivienne Turtle-Savage

**Affiliations:** Foot Ulcer Trials Unit, Department of Diabetes and Endocrinology, Nottingham University Hospitals NHS Trust, City Hospital Campus, Hucknall Road, Nottingham, NG5 1 PB UK; Cardiff University, Cardiff, UK; Swansea University, Swansea, UK; Nottingham Clinical Trials Unit, Nottingham, UK; Royal Derby NHS Foundation Trust, Derby, UK

**Keywords:** Diabetic foot ulcer, Heel, Pressure sore, Ulcer healing, Diabetes complications, Neuropathy, Casting, Off-loading, Amputation

## Abstract

**Background:**

Ulcers of the heel in diabetes are the source of considerable suffering and cost. In the absence of specific treatments, it has been suggested that removable, lightweight fibreglass heel casts may both promote healing and reduce discomfort and pain. The aim of the study is to assess the effectiveness and cost-effectiveness of fibreglass heel casts in the management of heel ulcers.

**Methods/Design:**

This is an observer-blind, randomised controlled trial in which participants with diabetes and heel ulcers (NPUAP/EPUAP grades 2, 3 or 4 and present for 2 or more weeks) are randomised to receive either usual care plus lightweight fibreglass heel casts or usual care alone. Randomisation is undertaken by random number sequence generation incorporated as part of the electronic case record form, and is stratified by both ulcer area (less than versus equal to or greater than 1 cm^2^) and NPUAP/EPUAP grade. Participants are followed every two weeks until healing or for 24 weeks. The primary outcome measure is healing at or before 24 weeks and maintained for 4 weeks. Secondary outcomes include (*i*) *ulcer-related outcomes*: time to healing, change in ulcer area, minor and major amputation, secondary infection and (*ii*) *patient-related outcomes*: local pain, mood and function (EQ-5D), impact of the ulcer (Cardiff Wound Impact Schedule) and survival. Cost-effectiveness will be assessed using a decision analytic model to estimate costs from the perspective of the UK NHS and personal social services and health outcomes, including percent healing and Quality Adjusted Life Years gained.

Safety will be documented as adverse and serious adverse device effects.

**Discussion:**

If it is possible to confirm significant clinical benefit and/or cost-effectiveness, this would have direct implications for the management of this distressing and costly complication of diabetes

**Trial registration number:**

ISRCTN62524796 Registered 29 March 2011

## Background

Chronic ulceration of some part of the foot affects up to 15% of all people with diabetes at some stage, and is the source of considerable cost and suffering[1]. The average age at presentation is 67 years and many of those affected are thus older aged, with significant co-morbidities and often clinging to an independent existence. Only two thirds of all ulcers heal without amputation within 12 months and in these, the median time to healing is 78 days[2, 3]. Forty percent of patients whose ulcers heal will develop a recurrence within 12 months[4].

Ulcers of the heel present particular difficulties and it has often been said traditionally that 'Heel ulcers don’t heal'. Seven percent of heel ulcers result in amputation of the limb in diabetes and 20% persist until death[5]. Despite this, a single centre review of a consecutive series of 154 heel ulcers in 97 patients with diabetes managed in UK revealed that the eventual incidence of healing of heel ulcers without surgery was very similar to ulcers elsewhere on the foot. The median time to healing was, however, very much longer at 200 (24 to 1,225) days[5] - almost 3 times longer than ulcers elsewhere on the foot. A recent multicentre survey in 14 centres in Europe reported that the median time to healing of heel ulcers was very similar, at 237 days[6]. Heel ulcers in diabetes also differ from ulcers elsewhere on the foot in that they are frequently painful.

Many heel ulcers arise as a result of the pressure of immobilisation, and pressure ulcers are very common in acute hospitals. Repeated prevalence surveys in UK suggest that 8 to 10% of all patients in acute hospital beds develop a pressure ulcer, of which one third are on the heel; half of all pressure ulcers are in people with diabetes. The prevalence of pressure ulcers is higher in long-stay hospitals and care homes (Pankhurst S, Clough A unpublished data).

While the principles of care have been specified by the National Institute for Clinical Excellence (NICE) and the Royal College of Nursing (RCN)[7], and by the International Working Group on the Diabetic Foot (International Diabetes Federation)[8], there are no specific interventions which have been shown to improve the outcome. The use of a non-removable below-knee fibreglass (total contact or variant) cast is known to hasten healing in ulcers caused by abnormal pressure loading on other parts of the foot[9], but it has been reported to be ineffective when the ulceration is on the heel[10].

In the absence of specific treatment of proven effectiveness in heel ulcers, a small number of specialists in UK advocated the use of lightweight, removable fibreglass heel casts, and produced uncontrolled observational evidence that these devices result in both a reduced time to healing and a prompt improvement in pain and discomfort. Thus, healing was observed in 42 (84%) of a consecutive series of 50 heel ulcers (in patients both with and without diabetes, but all of whom had peripheral arterial disease), with a median (range) time to healing of 6 (3 to 13) weeks[11]. Although the apparent benefit seems greater than would be expected for such a simple device, the findings of this uncontrolled study are mirrored by the clinical impression of the applicants. The mechanism for any positive effect is not known but may relate to the reduction of shearing and stretching forces applied to the surface of the ulcer. Current strategies to reduce local forces in an area of ulceration (or ulceration risk) are largely concentrated an attempt to reduce vertical forces with minimal effect, if any, on shear and on stretching.

Lightweight fibreglass heel casts take approximately 15 minutes to mould to the heel and can be easily fashioned in a domiciliary setting. They are applied over the primary wound dressing and held in place with an outer dressing, being saved and re-used each time the dressing is changed. They are replaced when stained, damaged or lost, and can often be worn inside shoes. Health care professionals can be trained in their use in approximately 30 minutes, and the material cost of each cast is approximately £7. Casts need to be replaced on average every three weeks.

The purpose of the proposed study is to systematically evaluate the effectiveness and cost implications of this simple and apparently beneficial intervention. The study was funded by NHS National Institute for Health Research, Health Technology Assessment Programme Grant 09/01/53.

## Methods/Design

### Study configuration

This will be an observer-blind, randomised controlled trial. Randomisation will be stratified by ulcer grade (National Pressure Ulcer Advisory Panel-European Pressure Ulcer Advisory Panel (NPUAP-EPUAP) Grade 2, 3 or 4, see below) and by ulcer area (greater than or equal to 25 mm^2^ and less than or equal to 1 cm^2^ or greater than 1 cm^2^), using random permuted blocks of randomly varying size. Randomisation will be undertaken by Nottingham Clinical Trials Unit (CTU), using a web-based system.

#### NPUAP-EPUAP ulcer grading system

1. Non-blanchable erythema
2. Partial thickness
3. Full thickness skin loss
4. Full thickness tissue loss
5. Unstageable/Unclassified: full thickness skin or tissue loss - depthunknown

#### Primary endpoint

The primary endpoint will be percentage of all ulcers healed at or before 24 weeks (6 months). Healing will be defined as epithelialisation maintained for 4 weeks and will be confirmed by an observer blind to the randomisation group.

#### Secondary endpoints

(i) *Ulcer-related outcomes*

Time to healing, change in ulcer area (measured by both acetate tracings and digital images analysed by using appropriate software to define area), infection, major and minor amputation, ulcer recurrence, secondary ulceration on either limb and adverse device effects (ADEs).

(ii) *Patient-related outcomes*

Local pain (visual analogue scale; VAS), Euroqol-5D (EQ-5D), Cardiff Wound Impact Schedule (CWIS), hospital admission (relating primarily either to the heel ulcer or not), hospital length of stay and death.

(iii) *Cost-effectiveness*

Cost-effectiveness will be assessed by developing a decision-analytic model to estimate costs and health outcomes, including percentage of healed ulcers and Quality Adjusted Life Years (QALYs) gained. Costs will be compared between the two groups using a bottom-up approach from the perspective of UK NHS and personal social services. Data relating to the costs of training professionals and the costs of heel casts and their application will be collected by discussions with relevant clinical and finance staff. Incremental cost-effectiveness and cost-utility ratios will be generated for a series of time horizons (including a lifetime perspective) that reflect management of patients with diabetic ulcers of the heel. The findings will be subjected to a series of one-way sensitivity analyses to determine the degree to which variation in parameter estimates affect the relative cost-effectiveness ratios. The results will be used to model the likely effects over the particular time horizons and costs and effects will be discounted at 3.5%. A probabilistic sensitivity analysis will be conducted to investigate the joint uncertainty in parameter values and cost-effectiveness acceptability curves will be generated. A budget impact analysis will be undertaken for 1-year and 5-year periods and will compare the costs to the health service of the use of heel casts in the management of ulcers of the heel in diabetes.

#### Safety endpoints

The study population is one which comprises older aged people who will have a high prevalence of co-morbidities, including renal, cardiac and cerebrovascular diseases. Moreover, ulcers of the foot may themselves worsen and lead to hospital admission because of infection or increasing necrosis. Although no specific safety issues are foreseen with the use of the heel cast, significant events are listed among the secondary outcome measures. Other unexpected ADEs will be recorded and if considered serious (SADE) will be reported to the sponsor in accordance with the principles of Good Clinical Practice (GCP).

The trial will be stopped when the last recruited subject reaches the end of the trial. However, premature termination of the clinical trial may occur because of a regulatory authority decision, change in opinion of the Research Ethics Committee (REC), or safety problems at the discretion of the Data Monitoring Committee (DMC) or Sponsor. No formal interim analyses for efficacy are planned by the Trial Management group (TMG). However, the DMC may need to assess efficacy in relation to safety in order to advise the Trial Steering Committee (TSC) appropriately. Stopping for safety will be based on an informal assessment by the DMC of adverse events. To aid interpretation of efficacy, Haybittle-Peto type boundaries of ± 3 standard errors (*P* < 0.0027) will be adopted to permit the DMC to break the blind with negligible effect on the properties of the final analysis.

Recruitment at any centre may be stopped for reasons including low recruitment and inadequate data recording.

### Randomisation and blinding

Internet-based treatment assignment will be determined by a computer-generated pseudo-random code using random permuted blocks of randomly varying size, created by the Nottingham CTU. Trial participants will be allocated with equal probability to each treatment arm with stratification by ulcer grade and ulcer size.

Participants will be randomised to be managed either with usual care recommended by the RCN and NICE[7] (see below) or with usual care plus a fibreglass heel cup. For participants randomised to the intervention group, a fibreglass heel cast will be made according to agreed procedures by clinical research staff who have been trained to an agreed level of competence. Participants or carers will be provided with written and verbal instructions on its use. Dressings may be changed as often as patients/carers feel necessary but this will be at least twice weekly.

#### Components of usual care

- Formal assessment of ulcer and surrounding skin
- Provision of any necessary off-loading
- Debridement (i) sharp, (ii) other as appropriate (but excluding the use of larvae)
- Appropriate dressing products
- Appropriate antibiotic therapy
- Nutrition and self care
- Optimal glycaemic control
- Revascularisation if deemed clinically necessary
- Continued close observation

### Study management

This trial will be conducted in accordance with independent ethics committee (IEC), relevant informed consent regulations (Declaration of Helsinki), ISO-14155 Guidelines and the Data Protection Act 1998. In addition all local regulatory requirements will be adhered to, in particular those which afford greater protection to the safety of trial subjects. All investigators and research staff will be fully trained in GCP, and the study will be conducted in line with these principles. There will be an independent DMC and a TSC and both will be constituted according to Medical Research Council (MRC) guidelines. The study has been approved by the Yorkshire & The Humber - Leeds West National Research Ethics Committee (reference 10/H1307/124) under the UK National Integrated Research Application Scheme. All participants gave written informed consent.

### Duration of the study and participant involvement

The start date was 1February 2011. Recruitment was planned to continue until 31 August 2014 and the intervention phase planned to end 28 February 2015. Recruitment was terminated on 4 September 2014 by which time 509 participants had been recruited.

#### Duration of participant involvement

If the ulcer remains unhealed, participants will have fortnightly assessments by the researcher and will remain in the study for 24 weeks. If the ulcer heals, they will have two more fortnightly assessments. If the ulcer remains healed, they will then have only their 12-week and 24-week assessments, unless these have already been done. If the ulcer heals at 22 or 24 weeks, the participant will have two further fortnightly assessments to confirm healing.

#### End of the study

The study will end when the final patient has completed their final study visit.

### Selection and withdrawal of participants

#### Recruitment

Patients will have diabetes associated with an ulcer (NPUAP-EPUAP grades 2, 3 or 4 - see Table 1) on the heel (that is affecting the skin below the malleoli but overlying the calcaneum inferiorly, posteriorly, medially or laterally) and which has been present for at least 2 weeks. Those affected will be identified by the health care professionals usually caring for them in hospitals, care homes or the community. The presence of peripheral arterial disease, wound infection and other particular co-morbidities (such as end-stage renal failure of immobilisation) will not be regarded as specific contraindications.

**Table 1:** **Fibreglass casts for heel ulcers in diabetes: trial profile**

	Screening visit	Visit 1 Randomisation	Visit 2	Visit 3	Visit 4	Visit 5	Visit 6	Visit 7	Visit 8	Visit 9	Visit 10	Visit 11	Visit 12	Visit 13
		Week 0	Week 2	Week 4	Week 6	Week 8	Week 10	Week 12	Week 14	Week 16	Week 18	Week 20	Week 22	Week 24
Check inclusion/exclusion criteria	**X**	**X**												
Information about study	**X**													
Informed consent	**X**													
Patient demographics		**X**												
ABPI		**X**												
Neuropathy		**X**												
Wound size by acetate tracing		**X**	**X**	**X**	**X**	**X**	**X**	**X**	**X**	**X**	**X**	**X**	**X**	**X**
Digital image after debridement		**X**	**X**	**X**	**X**	**X**	**X**	**X**	**X**	**X**	**X**	**X**	**X**	**X**
Cast applied if randomised to cast group		**X**	**X**	**X**	**X**	**X**	**X**	**X**	**X**	**X**	**X**	**X**	**X**	
Medication log		**X**	**X**	**X**	**X**	**X**	**X**	**X**	**X**	**X**	**X**	**X**	**X**	**X**
EQ-5D		**X**						**X**						**X**
CWIS		**X**						**X**						**X**
Pain VAS	**X**	**X**	**X**	**X**	**X**	**X**	**X**	**X**	**X**	**X**	**X**	**X**	**X**	**X**
Non-blinded Assessment			**X**	**X**	**X**	**X**		**X**	**X**	**X**	**X**	**X**	**X**	**X**
Collect health economic diary		**X**	**X**	**X**	**X**	**X**	**X**	**X**	**X**	**X**	**X**	**X**	**X**	**X**
Blinded assessment								**X**						**X**
							**Visit at which ulcer is judged healed**		**2 weeks after ulcer first judged healed**	**4 weeks after ulcer first judged healed**		**Visit 7 Week 12**		**Visit 13 Week 24**
Digital image after debridement							**X**		**X**	**X**		**X**		**X**
Cast applied if randomised to cast group							**X**		**X**	**X**		**X**		**X**
Medication log							**X**		**X**	**X**		**X**		**X**
EQ-5D												**X**		**X**
CWIS												**X**		**X**
Pain VAS							**X**		**X**	**X**		**X**		**X**
Non-blinded assessment							**X**		**X**	**X**		**X**		**X**
Collect health economic diary							**X**		**X**	**X**		**X**		**X**
Blinded assessment							**X**			**X**		**X**		**X**

If an ulcer is judged healed on or before Week 24, then healing should be confirmed by a blinded assessor as soon as possible, and within 4 days. If the ulcer breaks down within 4 weeks of first being judged healed, the participant should continue in the study. If the ulcer remains healed, the participant needs to have 12 Week and 24 Week visits – unless these have already been done. ABPI, ankle brachial pressure index; CWIS, Cardiff Wound Impact Schedule; EQ-5D, Euroqol-5D; VAS, visual analogue scale.

#### Screening log

A log will be kept at each centre which will list patients screened but not included in the study. The data kept in the log will include only the date of screening, patient initials, age and reason for screen failure. The log will not leave the clinical centre and the only data collected from the centre will be the absolute numbers of patients screened patients and the reasons for screen failure.

#### Inclusion criteria

- Type 1 or type 2 diabetes mellitus
- Age 18 years or over
- An ulcer of the heel (below the malleoli and affecting the skin overlying the calcaneum) of NPUAP-EPUAP Grade 2 to 4, which has been present for 2 or more weeks and which has a cross-sectional area ≥ 25 mm^2^. If there is more than one heel ulcer, one - which will be the largest or that judged most clinically significant - will be selected as the index ulcer
- Subjects who are both able and willing to give written informed consent

#### Exclusion criteria

- Frailty or disability which would mean that participation in the study might have an adverse effect on patient well-being and mood
- The need for any off-loading device to be non-removable
- The likelihood of protocol violation because of planned travel
- Those who withhold consent
- Active participation in another study of a wound care product
- The use of topical negative pressure or application of larvae to the index heel ulcer

### Study procedure

#### Study device

The study device is a fibreglass heel cast, moulded to the shape of the heel and split for easy removal and replacement. The heel cast will be made according to a study specific procedure and applied over the primary dressing (and secondary dressing if appropriate) which will be selected at the discretion of the patient’s usual carer. The cast will be covered by a single layer of Softbanor equivalent bandage and held in place by a retention layer. Written instructions on device usage will be given to patients and care givers. Heel casts will be replaced when worn or stained.

#### Study plan

Participants who give written informed consent will be asked either to attend a research clinic, or will have the necessary information and examination at another venue which is convenient for them and their carer.

The following procedures will be performed at the appropriate visit(s)

1. *Prior to randomisation visit*

The nature of the study will have been explained to the patient/carer by their usual doctor/nurse/podiatrist

2. *Randomisation visit 1* (*week 0*)

Check inclusion and exclusion criteria

Explain study to patient

Written informed consent/agreement

Documentation of demographics and baseline clinical details, including:

- Assessment of neuropathy (loss of protective sensation) using a 10 g monofilament applied to three sites (hallux, first and fifth metatarsal heads) on the sole of the index foot
- Assessment of peripheral arterial disease by palpation of pedal pulses and ankle brachial pressure index (ABPI)
- Ulcer grade by NPUAP/EPUAP
- Digital image of ulcer following any necessary debridement
- Local pain assessment by VAS
- Patient well-being and function documented using EQ-5D and Cardiff Wound Impact Schedule (CWIS)
- Documentation of medication
- Tracing of ulcer area with acetate sheet for the purposes of randomisation and stratification

#### Randomisation

3. *Visits 2to 13 will be every 2 weeks* (*±4 days*) *to 24 weeks* (*visit 13*)

During each visit a check will be made for healing. If the ulcer is judged healed, it will be confirmed by blinded a clinician within 4 days. It will then be reviewed after two weeks (±4 days) weeks and 4 weeks (±4 days). If the ulcer is thought to remain healed at 4 weeks, healing will be confirmed once more by a blinded clinician. If the ulcer recurs during this period, the participant should return to the intervention phase of the study. If the ulcer does not recur, the participant should continue to collect data for health economic analysis and should be reviewed by the research staff at 12 and 24 weeks, assuming these dates have not already passed.

Other measures to be taken at different visits are charted in Table 1. The wound will be cleaned, debrided (when appropriate), traced and imaged at each visit before being redressed. Patient well-being and function will be documented by completion of EQ-5D and CWIS (weeks 12 (±1) and 24 (±2) only.

For participants randomised to the intervention group, a fibreglass heel cast will be replaced as necessary by trained personnel and the patient/carer will be given written and verbal instructions on its use.

### Protocol violations

The following will be regarded as a protocol violation:

- Failure to use the study intervention (fibreglass heel cast) as recommended for more than 7 consecutive days, or for more than a cumulative total of 14 days during the course of the 24 week study. Those who violate the protocol in this way alone will not be withdrawn but will continue with fortnightly visits

The following will be regarded as a protocol violation necessitating withdrawal from the study:

- Omission of more than one consecutive scheduled fortnightly assessment by the researcher, or omission of a total of 3 or more of these visits during the 24 week study, or until healing is confirmed

### Withdrawals

A patient can be withdrawn from the study if:

- Consent/agreement is withdrawn
- The participant loses capacity
- It is found that they were recruited in error
- They have omitted more than one consecutive scheduled fortnightly assessment by the researcher, or omitted a total of three or more of these visits during the 24 week study, or before healing is confirmed
- The participant is lost to follow-up

### Assessment of safety

#### Adverse device effect (ADE)

This refers to any untoward and unintended response to a medical device, including any effect resulting from insufficiencies or inadequacies in the instructions for use or the deployment of the device and any effect that is a result of a user error. ADEs will be recorded as they are reported whether spontaneously volunteered or in response to questioning about well-being at trial visits. All ADEs will be documented in the subject’s medical records and CRF and will be followed until resolution, or for at least 30 days after discontinuation of the use of the device, whichever comes first. The investigator will assess causal relationship of the ADE to the investigational device according to the following classification:

- None: no relationship with investigational device. Other factor(s) certainly or probably causative
- Possible: reasonable possibility that the event was caused by the device, even though other possible causative factor(s) may exist
- Probable: the event was certainly or probably caused by the device, even though other possible causative factor(s) may exist

The following definitions for rating severity of ADEs may be used:

- Mild: signs or symptoms which are easily tolerated and are transient and only mildly irritating. There is no loss of time from normal activities and symptoms do not require medication or a medical evaluation
- Moderate: discomfort sufficient to cause interference with usual activities or require therapeutic intervention; for example, concomitant medication
- Severe: incapacity with inability to do work or undertake usual activities

#### Foreseeable ADEs

Foreseeable ADEs are those related to worsening of the clinical state of the ulcer and will be reported as secondary outcomes. These include

*Ulcer-related outcomes*

Increase in ulcer area

Infection

Major and minor amputation

Ulcer recurrence

Secondary ulceration on either limb

*Patient-related outcomes*

Increase in pain

Worsening mood or function

Hospital admission (relating primarily to the heel ulcer)

Death from pre-existing medical conditions

SADEs

An ADE that has resulted in any of the consequences characteristic of the serious criteria or that might have led to any of these consequences if suitable action had not been taken or if circumstances had been less opportune. These serious criteria are:

- Death
- Life threatening illness or injury
- Hospitalisation or prolongation of hospitalisation
- Permanent impairment of body structure or body function
- Medical or surgical intervention required to prevent any of the above

An unexpected SADE refers to any SADE, the specificity or severity of which is not consistent with the current protocol.

*SADE Reporting*

All SADEss must be reported immediately to the Sponsor and will be documented in the subject’s medical records and CRF. All SADEs must be followed until resolution, or for at least 30 days after discontinuation of device use, whichever comes first.

### Statistical analysis

Appropriate descriptive statistics will be presented for each variable. Two-sided statistical test will be performed and presented with exact *P*-values, with interval estimates constructed using 95% as the level of confidence.

Descriptive statistics, analytical methods and sample size considerations will be included. The study team together with the investigators will make the decisions regarding individual values belonging to a patient to be excluded from the analysis; for example, out of range values/values that cannot be objectively verified. The investigators will make the final decision regarding coding on the reason(s) for withdrawal. The safety population (SP) will comprise all randomised patients for the study

The intention-to-treat (ITT) population is defined as all subjects who were randomised. Given the study is based on a population with an expected high morbidity, the attrition rate will be monitored closely. If the missing primary outcome data fall within the 20 to 25% level, then ITT will be completed without imputation in the first instance and repeated with multiple imputation as a sensitivity analysis. ITT is equal to SP in this study. Once a participant has a healed wound, which is confirmed as ‘healed’, they will no longer participate in the intervention phase of the study, as they will have achieved the primary outcome. Such participants will be deemed to have completed their participation in the efficacy part of the study and their data will be included in the numbers ‘healed’ by 24 weeks.

Per protocol (PP) population is defined as a subset of ITT population that has completed the treatment period of maximum 24 weeks according to the protocol. The following will be regarded as a protocol violation necessitating exclusion from the PP analysis:

- Failure to use the study intervention (fibreglass heel cast) as recommended for more than 7 consecutive days, or for more than a cumulative total of 14 days during the course of the 24-week study
- Omission of more than one consecutive scheduled fortnightly assessment by the researcher, or omission of a total of 3 or more of these visits during the 24-week study

Statistical analyses of relevant safety and all efficacy variables will be performed on the ITT population. Additionally, statistical analyses of efficacy variables concerning ulcer measurements will be performed on the PP population. If ITT and PP analyses of efficacy produce similar results, only the ITT analysis will be presented in the report, and the PP analysis will briefly be summarised.

### Statistical methods

All assumed continuously distributed variables will be investigated with regards to distribution. Transformation to unconstrained scales for ulcer area and VAS for pain will be considered. All continuously distributed variables will be summarised by treatment at measured time points with n = number of subjects, mean, 95% confidence interval (CI) of mean, median, standard deviation (SD), minimum, and maximum. Change from the baseline to the end will also be summarised by treatment with n = number of subjects, mean, median, SD, 95% CI of mean, minimum and maximum. All categorical (discrete including ordinal) variables will be presented in contingency tables showing counts and percentages for each treatment group at all time points.

#### Demographic and other baseline variables

All demographic and diagnostic ulcer assessments at baseline will be presented descriptively with counts and rates. In addition, age will be given descriptively with n, mean, median, SD, minimum-maximum. These data will be presented by trial arm at baseline.

#### Primary outcome

Healing will be defined as epithelialisation maintained for 4 weeks and will be confirmed by an observer blind to randomisation group. Outcomes for all patients randomised will be presented using a CONSORT flow diagram.

*Classification of outcome will include the following rule set:*

1. Amputation of the target limb is a ‘failed’ outcome and classified as ‘not-healed’
2. For those who achieve healing during the intervention phase (and are confirmed as healed) but have an active ulcer at the 24-week assessment will be counted as ‘healed’ but the recurrence/new ulcer will be noted as a secondary outcome
3. For those who achieve healing during the intervention phase (and are confirmed as healed) but do not attend for the 24-week assessment will be counted as ‘healed’
4. If the missing primary outcome data fall within the 20 to 25% level, then ITT will be completed without imputation in the first instance and repeated with multiple imputation as a sensitivity analysis

The primary endpoint will be percentage of all ulcers healed at or before 24 weeks (6 months). Based on the binary nature of the outcome, the unadjusted analysis of the effect of treatment on the primary outcome will be assessed using Logistic Regression. Results will be reported as an odds ratios (OR), 95% CI and exact *P*-values.

A covariate adjusted analysis of the effect of treatment on the primary outcome will be undertaken. All prognostic variables and stratification variables at randomisation (ulcer area and NPUAP grade) will be eligible for inclusion in the covariate adjusted model. Prognostic variables shown to be strongly associated with outcome, even if not shown to be imbalanced between treatment groups, will be screened for inclusion in the covariate adjusted model as they may remove bias from the estimate of treatment effect.

Univariate logistic regression will be conducted to evaluate the relationship between each prognostic variable identified and the study primary outcome. Prognostic variables (and those with a strong imbalance at baseline) with a Likelihood Ratio Test (LRT) *P*-value less than or equal to 0.15 will qualify for evaluation in the maximum covariate adjusted model. Inferences will not be drawn from the interpretation of this univariate *P*-value, but will simply be used to describe the strength of association between the prognostic variable and the primary outcome.

All identified variables will be included in a maximum covariate adjusted logistical regression model. The treatment group term will be forced to stay in the maximum model; the model outcome will be the primary outcome. Using a backwards-stepwise elimination from the maximum model, the final covariate adjusted model will contain all prognostics and unbalanced variables known to have a meaningful bias on the estimate of the treatment effect. The LRT *P*-value for the estimate of treatment effect from this model will be reported, together will the covariate adjusted OR for treatment effect, and the appropriate 95% CI.

#### Secondary outcomes

Testing of the secondary outcome variables is exploratory and the results should mainly be used for exploratory purposes. On this basis, no adjustments for multiple testing will be performed.

The time to complete healing of the reference ulcer between the two randomized groups is an important secondary outcome and will be compared using survival analysis. Kaplan-Meier survival curves will be produced for the 2 groups and the median time to healing with 95% CI presented. Treatment effect will be explored using a Cox proportional hazards model including stratification factors (ulcer size and grade) initially. Participants’ heel ulcers that are not healed will be treated as censored and their date of trial exit, or date of last available assessment, or 168-day/trial cessation, as appropriate, will be used to calculate their duration in the trial. Hazard ratio with 95% CI will be given.

Final ulcer area will be analysed by treatment group for those who remain unhealed at the end of the study, using linear regression. As this exploratory analysis will include a subset of the participants, the analysis will be adjusting for baseline area, and any other variables that are found to be imbalanced at baseline (ANCOVA: on both an ITT and PP basis).

Ulcer related pain (measured on a VAS), will be analysed using linear regression and adjusting for baseline pain score, ulcer area, duration of ulcer, and ulcer type on both an ITT and PP basis.

Scores on standardised scales (EQ-5D) will be calculated in line with the guidelines provided by the original authors. The three domain scores will be calculated on the CWIS for each participant, in line with the standard instructions for use: descriptive statistics will be used to summarise these at each assessment. The standardised areas under the curve (area under the curve for each participant adjusted for the duration of available data) will be reported for both groups and compared using a Wilcoxon rank sum test.

*Secondary variables: Descriptive*

All other variables that will not be modelled formally will be considered descriptively. These are:

(i) *Concomitant medications*

All concomitant medications will be listed descriptively by study treatment.

(ii) Usual wound assessment and care requirements

Routine data related to the formal assessment of the wound and surrounding skin will be presented descriptively with counts and rates; provision of off-loading will be presented as categorical data with changes to device requirements recorded throughout the intervention period noted.

*Secondary variables: Safety*

ADEs and withdrawals comprise safety data and will be presented in line with standard reporting requirements. SADEs will also be considered and presented by group. Secondary outcome measures (incidence of foot infection, major amputation, minor amputation, revascularisation and falls leading to hospital admission) will be reported.

Using a negative binomial model and adjusting for the same covariates as the primary analyses, the numbers of adverse events in each participant between treatment groups will be compared.

#### Missing data

Due to the design of the electronic CRF, within which a missing field is not permitted, the amount of missing data within an assessment visit should be kept to a minimum. However, missing data could still occur due to failure to attend for an assessment visit. Missing primary outcome data will be investigated through multiple imputation as a secondary sensitivity analysis of the primary outcome, should the missing data fall within the 20to 25% range.

#### Determination of sample size

The expected prevalence of healed ulcer in the control group and the treatment group is 40% and 55%, respectively, at 24 weeks. With α = 0.05 and β = 1-power (power 80%) and an estimated rate of dropouts of 30% a total of 531 patients of both genders will be included (to achieve 186 in each group completing the intervention phase). With 25% attrition, the number required is 496 (to achieve 186 in each group). The study will aim to recruit 500 patients and closely monitor the number of cases where the primary outcome data are missing. All secondary analyses will be interpreted with caution as the sample size calculation is based on the primary outcomes only. Internet-based treatment assignment will be determined by a computer-generated pseudo-random code using random permuted blocks of randomly varying size, created by the Nottingham CTU. Trial participants will be allocated with equal probability to each treatment arm with stratification by ulcer grade and ulcer size.

## Discussion

Ulcers of the heel constitute a major source of morbidity and cost, and there has hitherto been no specific therapy described. Recently, however, the use of lightweight fibreglass heel casts has been described and has been reported to improve both the incidence and speed of healing. The purpose of this trial is to document the efficacy of this intervention, as well as its cost, when compared to the use of defined criteria of best usual care.

The trial is randomised and while it is not possible to blind either the participant or their carer to the allocated arm of the study, the primary outcome measure (clinical healing) will be confirmed by a blinded observer. All non-clinical measures (including digital images of ulcer, measures of pain and discomfort) will be undertaken by blinded observers.

The study population is designed to be as inclusive as possible, with the aim of maximising the external validity of any findings. Thus, the intention is that as large a percentage as possible of people with diabetes complicated by ulceration of the heel could be included - including those with active infection, as well as those with all but the most severe peripheral arterial disease. This population is, however, one with multiple co-morbidities, a high expected incidence of unrelated illness and hospital admission and a poor overall life-expectancy. It is for this reason that a decision has been made to restrict safety measures to device effects. It is also for this reason that the sample size calculation allows for up to 30% attrition, and this is based on previous experience gained in conducting trials in this area.

While the primary outcome measure is that of healing, the trial includes a number of secondary outcome measures. Principal among these are rate of change in ulcer area - as an indication of the likelihood of eventual healing, and the experience of local pain and discomfort. While most foot ulcers in diabetes are typically painless, those on the heel are often the source of pain or other discomfort and experience reported by those who have used such casts in clinical practice is that reduction in local discomfort is often observed.

## Trial status

Recruitment started on 20 April 2011 and ceased on 4 September 2014 by which time 509 participants had been randomised. A total of 35 centres were involved throughout the UK, but recruitment was discontinued at 8 because of poor recruitment and/or poor documentation.

## Abbreviations

ABPI: ankle brachial pressure index
ADE: adverse device effect
CI: confidence interval
CRF: case report form
CTU: clinical trials unit
CWIS: Cardiff Wound Impact Schedule
DMC: Data Monitoring Committee
EPUAP: European Pressure Ulcer Advisory Panel
EQ-5D: Euroqol-5D
GCP: Good clinical practice
ITT: intention-to-treat
LRT: Likelihood Ratio Test
MRC: Medical Research Council
NHS: National Health Service
NICE: National Institute for Clinical Excellence
NIHR: National Institute for Health Research
NPUAP: National Pressure Ulcer Advisory Panel
OR: odds ratio
PP: per protocol
QALYs: Quality Adjusted Life Years
RCN: Royal College of Nursing
REC: Research Ethics Committee
SADE: serious adverse device effect
SD: standard deviation
SP: safety population
TMG: Trial Management Group
TSC: Trial Steering Committee
VAS: visual analogue scale
